# P-657. Clinical Characteristics and Microbiology of Patients with Pneumonia after Battlefield Trauma

**DOI:** 10.1093/ofid/ofaf695.870

**Published:** 2026-01-11

**Authors:** Luke Jackson, Laveta Stewart, M Leigh Carson, Erica Sercy, Wesley Campbell, Andrew Wyatt, Katrin Mende, David R Tribble, John Kiley

**Affiliations:** Brooke Army Medical Center, San Antonio, TX; Infectious Disease Clinical Research Program, Henry Jackson Foundation, Bethesda, Maryland; Uniformed Services University of the Health Sciences & Henry M. Jackson Foundation for the Advancement of Military Medicine, Bethesda, Maryland; Infectious Disease Clinical Research Program, Department of Preventive Medicine and Biostatistics, Uniformed Services University of the Health Sciences; Henry M. Jackson Foundation for the Advancement of Military Medicine, Inc., Washington, District of Columbia; Walter Reed National Military Medical Center, Bethesda, Maryland; Landstuhl Regional Medical Center, Landstuhl, Rheinland-Pfalz, Germany; Infectious Disease Clincial Research Program, JBSA Ft Sam Houston, Texas; Uniformed Services University of the Health Sciences, Bethesda, Maryland; BAMC, San Antonio, Texas

## Abstract

**Background:**

Lower respiratory infections are a major contributor to morbidity after battlefield trauma. Between 2009-10, 8.5% of 423 US military casualties developed pneumonia (PNA) with a higher proportion (18.5%) in patients admitted to ICUs. Using a larger population, we examined characteristics associated with PNA after battlefield trauma.

**Figure:**
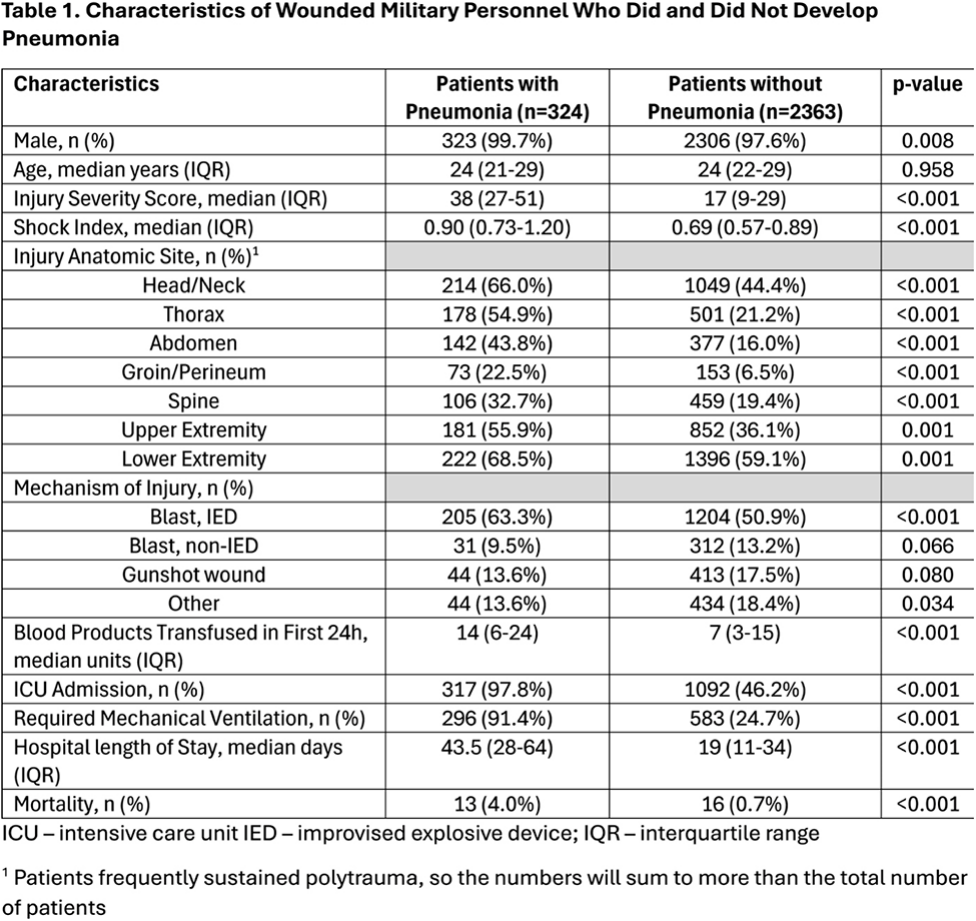
Characteristics of Wounded Military Personnel Who Did and Did Not Develop Pneumonia ICU – intensive care unit IED – improvised explosive device; IQR – interquartile range 1 Patients frequently sustained polytrauma, so the numbers will sum to more than the total number of patients

**Figure:**
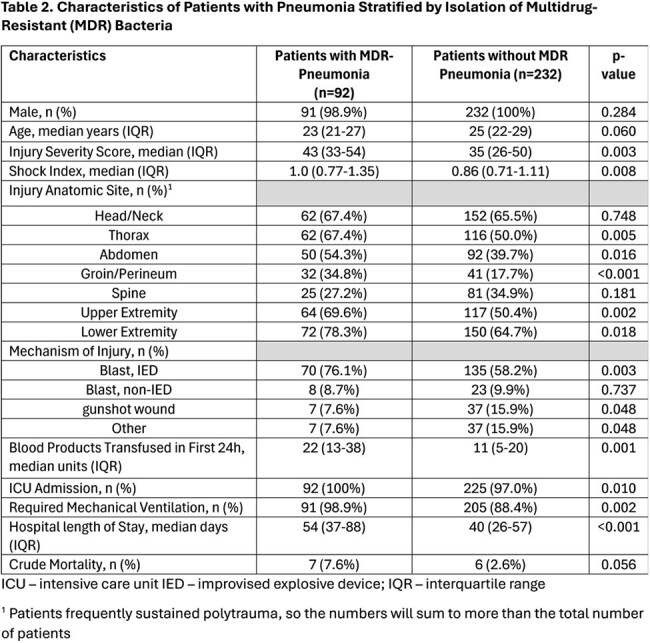
Characteristics of Patients with Pneumonia Stratified by Isolation of Multidrug-Resistant (MDR) Bacteria ICU – intensive care unit IED – improvised explosive device; IQR – interquartile range 1 Patients frequently sustained polytrauma, so the numbers will sum to more than the total number of patients

**Methods:**

Data were collected via the Trauma Infectious Disease Outcomes Study, an observational study of infections in wounded US military personnel (2009-2014). PNA was defined using standardized criteria. Chi-square (or Fisher exact) and Mann-Whitney U tests were used for categorical and continuous variables, respectively.

**Figure:**
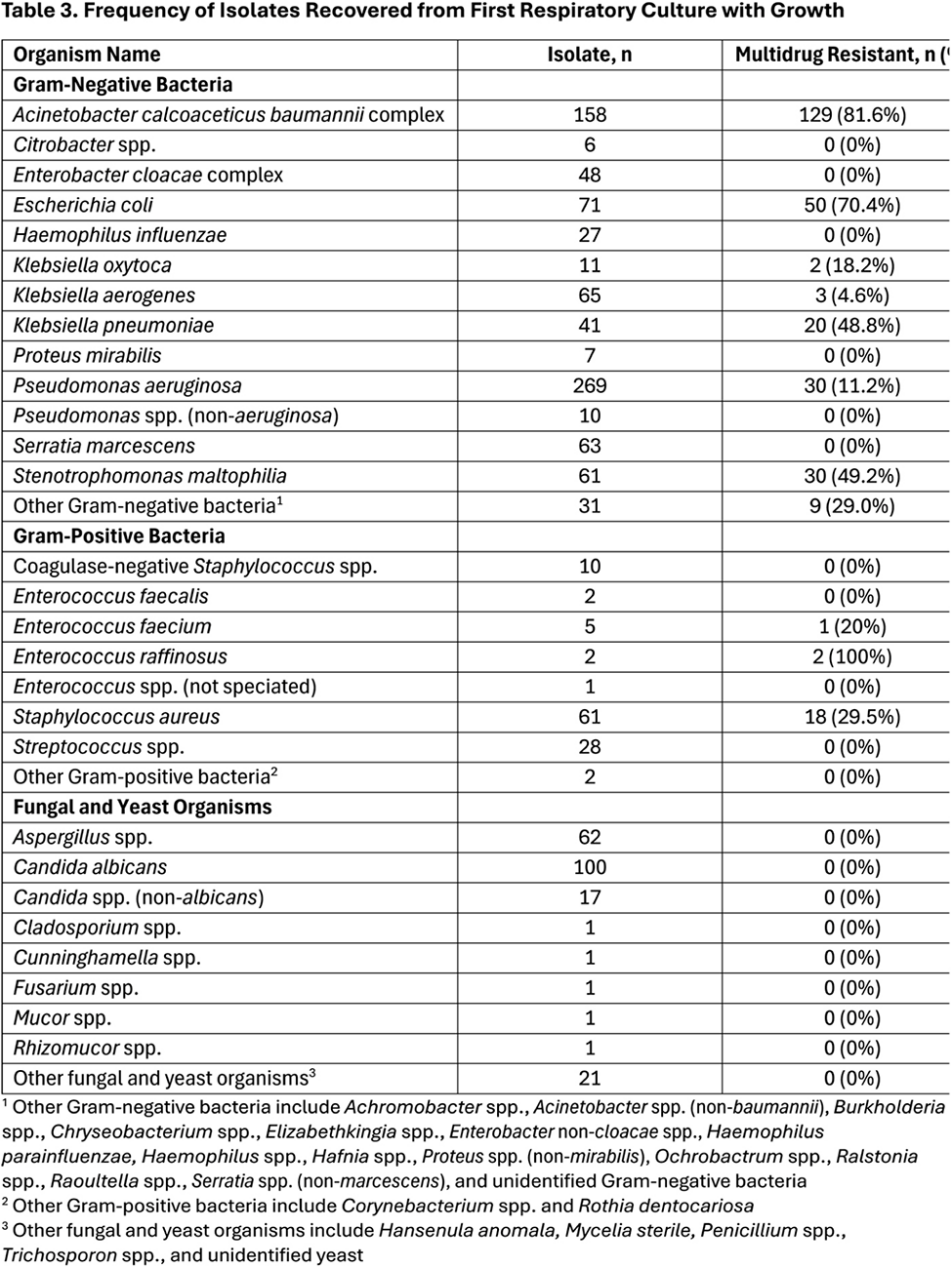
Frequency of Isolates Recovered from First Respiratory Culture with Growth 1 Other Gram-negative bacteria include Achromobacter spp., Acinetobacter spp. (non-baumannii), Burkholderia spp., Chryseobacterium spp., Elizabethkingia spp., Enterobacter non-cloacae spp., Haemophilus parainfluenzae, Haemophilus spp., Hafnia spp., Proteus spp. (non-mirabilis), Ochrobactrum spp., Ralstonia spp., Raoultella spp., Serratia spp. (non-marcescens), and unidentified Gram-negative bacteria 2 Other Gram-positive bacteria include Corynebacterium spp. and Rothia dentocariosa 3 Other fungal and yeast organisms include Hansenula anomala, Mycelia sterile, Penicillium spp., Trichosporon spp., and unidentified yeast

**Results:**

A total of 2,687 wounded military personnel were assessed, with 324 (12%) patients developing PNA. The PNA patients had higher injury severity scores (ISS; median 38 vs 17; p< 0.001) with more injuries to the head/neck (66% vs 44%), thorax (55% vs 21%), abdomen (44% vs 16%), groin/perineum (23% vs 7%), spine (33% vs 19%), upper extremities (56% vs 36%), and lower extremities (69% vs 59%) than non-PNA patients (p≤0.001, Table 1). PNA patients also had more ICU admissions (98% vs 46%), greater mechanical ventilation requirements (91% vs 25%), longer hospitalizations (median 44 vs 19 days) and higher crude mortality 4% vs 0.7% (p< 0.001). Among PNA patients, 92 (28%) had multidrug-resistant (MDR) bacteria isolated and the MDR PNA group had greater median ISS (43 vs 32 p=0.003), blood units in 1^st^ 24 hours of injury (median 22 vs 13 p=0.001), ICU admissions (100% vs 97% p=0.010), mechanical ventilation (99% vs 88% p=0.002), and hospital stays (median 54 vs 40 days p< 0.001) than PNA patients with non-MDR bacteria (Table 2). Most frequent bacteria were *Pseudomonas aeruginosa* (N = 269, 11% MDR) and *Acinetobacter baumannii* (N = 158, 82% MDR, Table 3).

**Conclusion:**

Battlefield-injured patients who developed PNA had greater injury severity along with more frequent injuries to the torso than non-PNA patients. PNA patients with MDR isolates were more severely injured with higher crude mortality than those with susceptible pathogens. These data support developing interventions to prevent PNA in these patients. Further analysis of clinical factors to include imaging and symptom reporting is planned.

**Disclosures:**

All Authors: No reported disclosures

